# Serum phosphatidylinositol depletion associates with fecal calprotectin and disease severity in female and male IBD patients

**DOI:** 10.1186/s12944-026-02889-3

**Published:** 2026-02-04

**Authors:** Hauke Christian Tews, Muriel Huss, Tanja Elger, Gerhard Liebisch, Marcus Höring, Johanna Loibl, Arne Kandulski, Martina Müller, Christa Buechler

**Affiliations:** 1 Department of Internal Medicine I, Gastroenterology, Hepatology, Endocrinology, Rheumatology, and Infectious Diseases, University Hospital Regensburg, 93053 Regensburg, Germany; 2 Institute of Clinical Chemistry and Laboratory Medicine, University Hospital Regensburg, 93053 Regensburg, Germany

**Keywords:** Phospholipid, Inflammatory bowel disease, Disease localisation, Disease severity, Ulcerative colitis, Crohn´s disease

## Abstract

**Background:**

Phosphatidylinositol (PI) is a phospholipid that exerts anti-inflammatory effects when injected during experimental colitis. The levels of PI species in the serum of patients with inflammatory bowel disease (IBD) and their association with disease activity have not yet been determined. This exploratory study investigates whether the levels of PI species in the serum are associated with the severity of the disease.

**Methods:**

Serum concentrations of 14 PI species were assessed using direct flow injection analysis with a triple quadrupole mass spectrometer. The study involved 16 healthy controls and 57 patients (including 26 females and 31 males) diagnosed with IBD.

**Results:**

Similar levels of all PIs measured were exhibited by patients with IBD and controls. Nine PI species were found to be higher in female patients, prompting a sex-specific analysis. Almost all PI species exhibited a negative correlation with fecal calprotectin in both sexes. Negative correlations of PI species with CRP were mostly found in males. PI 34:1, 36:1, 36:2, and 40:5 were significantly reduced in male and female patients with active disease compared to those with quiescent disease. In the entire patient cohort, all PI species significantly declined in active disease compared to patients with inactive disease and compared to healthy controls. Serum PI species levels were not associated with disease localisation in patients with Crohn’s disease, but were increased in ulcerative colitis patients with proctosigmoiditis in comparison to patients with pancolitis. The levels of PI 38:4, 38:5, 40:4, and 40:5 were higher in the serum of patients with Crohn’s disease than in those with ulcerative colitis, despite having similar disease activity.

**Conclusions:**

This study demonstrates that serum PI species decline in active IBD in both sexes. Specific PI species may evolve new biomarkers to discriminate patients with Crohn´s disease from patients with ulcerative colitis.

**Supplementary Information:**

The online version contains supplementary material available at 10.1186/s12944-026-02889-3.

## Introduction

Phosphatidylinositol (PI) is a minor phospholipid found in mammalian cells and human serum [1]. PIs are converted into phosphorylated derivatives, such as phosphatidylinositol-3,4,5-trisphosphate (PIP3) by PI3-kinase, thereby activating different signalling pathways [1]. PIP3 activates the protein kinase Akt, which plays a role in vital cellular processes such as insulin signalling and carcinogenesis [2, 3]. The PI3-kinase/Akt pathway is also important for tumor necrosis factor activation of nuclear factor kappa B [4].

The study of lipids in clinical samples, known as lipidomics, has emerged as a method to quantify hundreds of lipids simultaneously. The potential of this method lies in its ability to identify biomarkers for disease diagnosis and monitoring, and to evaluate the role of different lipids in disease pathophysiology [5–8]. Most lipids in the circulation are carried by lipoprotein particles, such as low-density lipoprotein (LDL) and high-density lipoprotein (HDL) [9]. PI supplementation in normolipidemic patients increased the cellular cholesterol levels of HDL particles [10]. PI enhanced reverse cholesterol transport, stimulating cholesterol efflux from peripheral cells and hepatic clearance [11, 12]. This may protect against hypercholesterolemia and atherosclerotic disease [11]. High LDL and low HDL levels are associated with metabolic diseases such as obesity and cardiovascular disease [13], and dietary PI may improve the clinical course of these common diseases.

Notably, LDL and HDL levels decline in chronic inflammation, and these patients have low serum cholesterol levels [14]. Patients with inflammatory bowel disease (IBD) also have reduced serum cholesterol during active disease phases [15–17].

Inflammation affects cholesterol biosynthesis and metabolism [18], but the pathways that contribute to hypocholesterolemia during inflammation are not fully understood. Studies have shown that the serum of patients with active IBD has a reduced capacity to stimulate cholesterol efflux from peripheral cells compared to the serum of healthy controls. This may result in cellular cholesterol accumulation and low serum cholesterol levels [16]. Furthermore, the depletion of cholesterol in Hep3B cells reduced their PI levels, particularly those of PI 36:2, which recovered upon cholesterol supplementation [19], demonstrating the close relationship between cholesterol and PI homeostasis.

PI in serum is carried by lipoprotein particles [12], which are decreased in chronic inflammation [14], but the association of PI with inflammation has received less attention. Macrophages obtained from the peripheral blood mononuclear cells of patients with Crohn´s disease (CD) synthesized less PI 16:0/18:1 than cells from healthy controls. The level of this PI species was also reduced in ileal biopsies of patients with CD [20]. Horta et al. were interested in identifying plasma biomarkers in IBD patients with fatigue, as this further impairs their quality of life. They described a decline in PI 14:0/18:1 levels compared to non-fatigued patients [21]. Thus, there is preliminary evidence that specific PI species are altered in IBD.

IBD is a chronic inflammatory disease of unknown aetiology, characterised primarily by CD and ulcerative colitis (UC) [22, 23]. C-reactive protein and fecal calprotectin are used clinically to monitor disease activity in CD and UC [24, 25]. However, neither of these markers is specific for IBD, and additional biomarkers for early diagnosis and disease activity monitoring are still being sought [24–27].

Despite evidence that PI plays a role in cholesterol homeostasis, which is disturbed in patients with active IBD [15–17, 28], to our knowledge, serum PI levels in patients with quiescent and active IBD have not been compared. It should be noted that the injection of PI during colitis induced by 2,4,6-trinitrobenzene sulfonic acid reduced T cell inflammatory response and colitis activity [29] further indicating that analysis of PI species in blood of patients with IBD may be of clinical interest.

### Hypothesis and study objective

Overall, studies analysing PI levels in IBD are rare; however, the available evidence suggests that individual PI species may exert distinct effects in IBD pathophysiology [20, 21].

To our knowledge, a comprehensive analysis of serum PI species levels in patients with IBD has not yet been reported. We hypothesise that serum levels of specific PI species differ between patients with IBD and healthy controls, and that these differences are associated with disease activity and lipid metabolism abnormalities, such as hypocholesterolemia. The objective of this explorative study is to quantitatively analyse 14 PI species in the serum of patients with IBD and healthy controls to (i) identify disease-associated alterations in PI profiles, and (ii) explore associations with disease severity and hypocholesterolemia.

## Materials and methods

### Patients and controls

Inpatients and outpatients admitted to a German university hospital between 12 June 2021 and 23 February 2023 were invited to participate in this study. Crohn’s disease (CD) and ulcerative colitis (UC) were diagnosed using endoscopic, histological, and clinical criteria [30, 31]. Individuals were excluded from the study if they had coagulopathy, were pregnant, or were unable to provide informed consent. Patients with primary sclerosing cholangitis were not included in this analysis. The control group consisted of the patients’ spouses, hospital staff and students. All participants signed written informed consent prior to enrolment. The study received approval from the Ethics Committee of the University Hospital Regensburg (protocol no. 19-1309-101; approval date: 20/02/2019) and was conducted in accordance with the latest good clinical practice guidelines and the updated Declaration of Helsinki.

### Lipid extraction and analysis of PI species

Lipids were isolated from 10 µL of serum samples following the protocol of Bligh and Dyer [32]. Non-naturally occurring PI 15:0/18:1[D7] (Avanti Polar Lipids, AL, USA) was added as an internal standard before lipid extraction. For extraction, a total volume of 2 ml of chloroform was used. Subsequently, 1 ml of the chloroform phase was separated, vacuum-dried, and dissolved in a methanol (Merck, Darmstadt, Germany)/chloroform (Roth, Karlsruhe, Germany) mixture (3:1, v/v) containing 7.5 mM ammonium acetate [33]. Lipid analysis was performed using direct flow injection analysis (FIA) with a triple quadrupole mass spectrometer (FIA-MS/MS, QQQ) operated in positive ion mode using the setup described previously [33]. A neutral loss of 277 was used to quantify PI species [34]. Data analysis involved correction of Type-II isotopic overlap [33] and Type-I isotopic effects [35]. Quantification was achieved based on the amount of the internal standard. Lipid species were classified in accordance with the recently released guidelines for shorthand notation of lipid structures obtained from mass spectrometry [36]. Further details of the workflow can be found in the lipidomics checklist [35], which is available as additional file 1.

### Statistical analysis

Data are presented as boxplots. Normality of PI species distributions was assessed using the Kolmogorov–Smirnov test. Most PI species were normally distributed (*P* > 0.05), except for PI 36:1 (*P* = 0.015) and PI 40:6 (*P* < 0.001). Depending on data distribution, comparisons were performed using one-way ANOVA with post hoc Dunnett’s test (for comparison of three or more groups), the Mann-Whitney U test, or the Kruskal–Wallis test with post hoc Bonferroni test. Correlations were analysed using Pearson’s or Spearman’s method, as appropriate. Receiver operating characteristic curves and multiple linear regression analysis were also performed. All analyses were conducted using IBM SPSS Statistics version 26.0 (IBM Corp., Armonk, NY, USA, 2019). A *P* value < 0.05 was considered statistically significant. In this exploratory study examining an unspecified topic, it is not advisable to adjust for multiple comparisons [37], and the *P*-values were not corrected for multiple comparisons. Hedges’ g effect sizes were calculated from means and standard deviations (www.socscistatistics.com/effectsize/). Cohen’s guidelines were utilized to interpret the effect size, with Hedges’s g values of 0.20, 0.50, and 0.80 being classified as small, medium, and large effects, respectively [38].

## Results

### Study cohorts

This study included 57 patients with inflammatory bowel disease (IBD), 26 females and 31 males (see Table 1 for details). Of the 57 patients 17 had ulcerative colitis (UC) and 40 patients suffered from Crohn´s disease (CD). Eleven women and five men participated as healthy controls, with no significant differences in sex distribution between cases and controls (*p* = 0.087). The control group had an average age of 52 years (range 24–78 years), which was higher compared to the age of the patients (*p* = 0.003). All controls were in good health and maintained a normal body mass index (BMI). However, no clinical laboratory measurements were documented for the controls.

**Table 1:** Baseline characteristics of the patient cohort. Details of the entire cohort of patients with inflammatory bowel disease (IBD), and of female and male cases. The median, minimum, and maximum values are shown. Statistical test: One-way ANOVA for age, aminotransferases, alkaline phosphatase and bilirubin; Mann-Whitney U test for body mass index, C-reactive protein, fecal calprotectin and gamma-glutamyl transferase

Characteristics	All Cases	Female Cases	Male Cases
Number	57	26	31
Age (years)	41.2 (19.1–69.9)	37.9 (19.1–69.9)	45.2 (20.0–66.9)
Body mass index (kg/m^2^)	24.1 (15.5–44.3)	22.8 (17.5–44.3)	24.8 (15.5–40.4)
C-reactive protein (mg/L)	2 (0–144)	2 (0–44)	2 (0–144)
Fecal calprotectin (µg/g)	58 (0–1616)	56 (0–1097)	78 (17–1616)
Aspartate aminotransferase (U/L)	25 (10–35)	22 (10–34)	26 (12–35)
Alanine aminotransferase (U/L)	18 (7–63)	17 (7–45)	20 (9–63)
Gamma-glutamyl transferase (U/L)	23 (8–100)	18 (8–37)	28 (11–100) ^*P* < 0.001^
Alkaline phosphatase (U/L)	65 (38–142)	59 (38–117)	69 (46–142)
Bilirubin (mg/dL)	0.4 (0.1–1.9)	0.4 (0.1–1.9)	0.5 (0.1–1.9)

No sex-specific differences were observed except for gamma-glutamyl transferase (GGT) levels, which were higher in male patients (Table 1).

### Serum PI species of patients and controls

The serum levels of 14 PI species were measured in patients with IBD and in healthy controls. There were no significant differences in the levels of these PI species or total PI levels between patients and controls (Table 2).

**Table 2:** Median, minimum and maximum serum PI levels of controls and patients with IBD. There were no significant differences between these groups. Statistical test: One-way ANOVA or Mann-Whitney U test (for PI 36:1 and 40:6)

PI nmol/ml	Control (*N* = 16)			IBD (*N* = 57)		
	Median	Minimum	Maximum	Median	Minimum	Maximum
34:1	3.34	2.37	11.55	3.16	0.96	7.47
34:2	1.98	1.24	5.44	2.04	0.69	4.22
36:1	3.76	2.24	8.31	4.22	0.94	7.09
36:2	8.38	5.27	20.27	8.19	2.49	15.42
36:3	1.79	1.04	4.07	1.81	0.45	3.49
36:4	2.94	1.83	5.94	2.97	0.84	6.81
38:2	0.34	0.15	0.65	0.36	0.11	1.00
38:3	6.23	3.67	8.76	5.52	1.20	16.82
38:4	35.72	20.22	57.89	38.29	10.51	79.54
38:5	2.35	1.18	4.42	2.26	0.37	5.36
38:6	0.59	0.32	1.11	0.42	0.10	1.32
40:4	0.57	0.22	0.90	0.56	0.12	1.50
40:5	1.47	0.52	2.13	1.18	0.25	3.02
40:6	1.65	0.77	3.25	1.38	0.36	4.84
Total PI	75.36	43.54	118.43	72.84	20.61	143.92

**Fig. 1:**
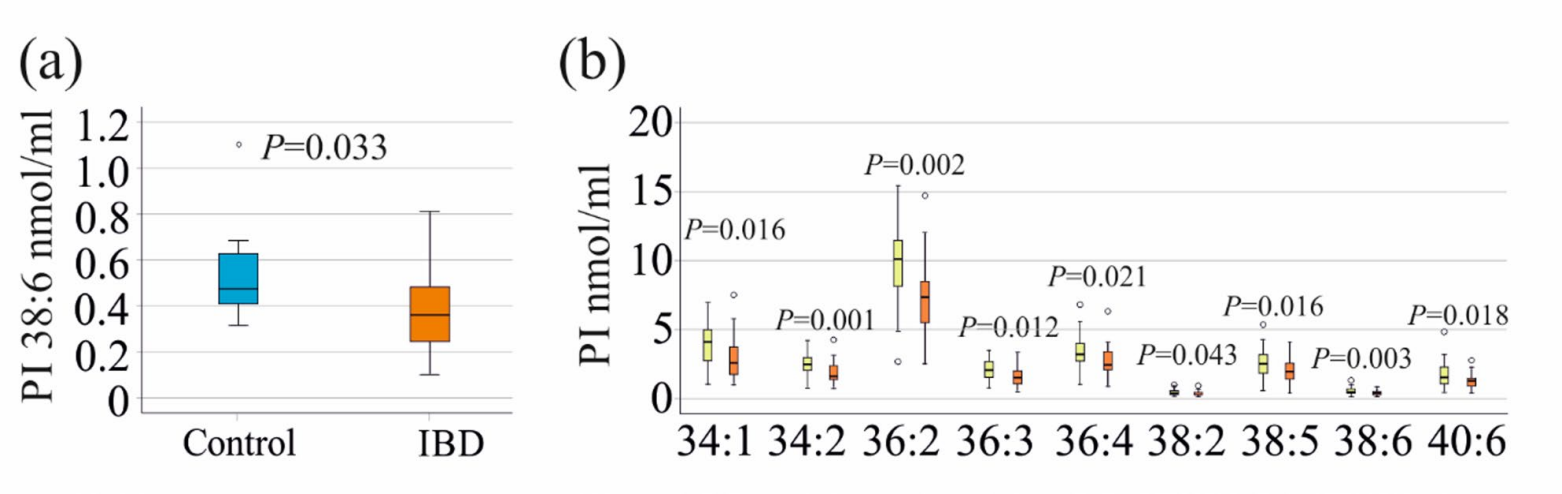
PI 38:6 of male controls and male patients, and sex-specific differences of PIs in IBD. **a** PI 38:6 in the serum of 5 male controls and 31 male patients with IBD. **b** PI species, which were increased in the serum of the 26 female (yellow boxes) compared to the 31 male patients (orange boxes) with IBD. Statistical Test: One-way ANOVA for all but PI 36:1 and PI 40:6, where Mann-Whitney U-test was used. The *P*-values in the figures were not corrected for multiple comparisons

The sex-specific comparison of serum PI species levels between patients and controls showed that PI 38:6 levels were significantly reduced in male patients compared to male controls (Fig. 1a). No significant differences in PI species were observed between female patients and controls (*P* > 0.05 for all).

### Associations of PI species with sex, age and BMI

The PI 36:3 (*P* = 0.019) and PI 38:5 (*P* = 0.004) species were found to be increased in the serum of female controls compared to male controls. There was no correlation between PI species and age of the healthy controls (*P* > 0.05 for all). However, in the case group, several PI species exhibited positive correlations with age, including PI 34:1 (*r* = 0.294, *p* = 0.027), 36:1 (*r* = 0.391, *P* = 0.003), 36:2 (*r* = 0.324, *P* = 0.014), 36:3 (*r* = 0.290, *P* = 0.029), 38:2 (*r* = 0.268, *P* = 0.044), 38:3 (*r* = 0.313, *P* = 0.018), 38:4 (*r* = 0.288, *P* = 0.030), 40:4 (*r* = 0.331, *P* = 0.012), and 40:5 (*r* = 0.346, *p* = 0.008). PI 36:1, 36:2, 36:4, 38:3, and 40:5 positively correlated with age in females, and PI 34:1, 36:1, 36:3 and 38:2 in males. Additionally, females had significantly higher levels of several PI species than males. These were PI 34:1, PI 34:2, PI 36:2, PI 36:3, PI 36:4, PI 38:2, PI 38:5, PI 38:6 and PI 40:6 (Fig. 1b). Total PI levels in the serum of females were increased compared to male cases (*P* = 0.025). A correlation between PI 40:4 and BMI (*r* = 0.311, *P* = 0.025) was found in the entire patient cohort.

There was no correlation of age with CRP (*r* = 0.040, *P* = 0.775) or fecal calprotectin (*r* = -0.235, *P* = 0.075).

### Correlation of phosphatidylinositol species with C-reactive protein, fecal calprotectin, and sex-specific subgroup analysis

In the entire cohort, all PI species and total PI levels showed a negative correlation with C-reactive protein (CRP) and fecal calprotectin (Table 3). In females, the PI species 36:1, 36:3, 40:5, and 40:6 were negatively correlated with CRP, while all PI species were negatively correlated with fecal calprotectin, except for PI 38:2 and PI 40:6. For male participants, all PI species except PI 34:2, 38:6, and 40:6 exhibited a negative correlation with both CRP and fecal calprotectin. Additionally, PI 38:6 and 40:6 were negatively correlated with CRP in males (Table 3).

**Table 3:** Correlation coefficients between PI species and C-reactive protein (CRP), as well as fecal calprotectin, in the entire cohort, in female and male cases. Statistical test: Pearson’s correlation was used for all but PI 36:1 and 40:6, for which Spearman’s correlation was analyzed. The *P*-values in the table were not corrected for multiple comparisons

PI Species	CRP	Fecal Calprotectin	CRP	Fecal Calprotectin	CRP	Fecal Calprotectin
	Entire Cohort (*N* = 57)		Females (*N* = 26)		Males (*N* = 31)	
34:1	-0.368 ^*P*=0.006^	-0.486 ^*P* <0.001^	-0.360	-0.504 ^*P*=0.009^	-0.371 ^*P*=0.043^	-0.459 ^*P*=0.009^
34:2	-0.339 ^*P* =0.012^	-0.422 ^*P*=0.001^	-0.307	-0.526 ^*P*=0.006^	-0.347	-0.346
36:1	-0.524 ^*P*<0.001^	-0.522 ^*P* <0.001^	-0.461 ^*P*=0.023^	-0.471 ^*P*=0.015^	-0.592 ^*P*=0.001^	-0.533 ^*P*=0.002^
36:2	-0.456 ^*P*=0.001^	-0.545 ^*P* <0.001^	-0.376	-0.584 ^*P*=0.002^	-0.511 ^*P*=0.004^	-0.534 ^*P*=0.002^
36:3	-0.435 ^*P*=0.001^	-0.524 ^*P* <0.001^	-0.437 ^*P*=0.033^	-0.477 ^*P*=0.014^	-0.457 ^*P*=0.011^	-0.539 ^*P*=0.002^
36:4	-0.363 ^*P*=0.007^	-0.427 ^*P* =0.001^	-0.250	-0.472 ^*P*=0.015^	-0.425 ^*P*=0.019^	-0.398 ^*P*=0.026^
38:2	-0.334 ^*P*=0.014^	-0.427 ^*P* =0.001^	-0.307	-0.337	-0.382 ^*P*=0.037^	-0.491 ^*P*=0.005^
38:3	-0.397 ^*P*=0.003^	-0.480 ^*P* <0.001^	-0.358	-0.413 ^*P*=0.036^	-0.422 ^*P*=0.020^	-0.498 ^*P*=0.004^
38:4	-0.435 ^*P*=0.001^	-0.477 ^*P* <0.001^	-0.254	-0.471 ^*P*=0.015^	-0.531 ^*P*=0.003^	-0.487 ^*P*=0.005^
38:5	-0.444 ^*P*=0.001^	-0.490 ^*P* <0.001^	-0.386	-0.462 ^*P*=0.017^	-0.557 ^*P*=0.001^	-0.533 ^*P*=0.002^
38:6	-0.373 ^*P*=0.005^	-0.350 ^*P*=0.008^	-0.384	-0.410 ^*P*=0.037^	-0.415 ^*P*=0.023^	-0.304
40:4	-0.436 ^*P*=0.001^	-0.503 ^*P* <0.001^	-0.253	-0.390 ^*P*=0.049^	-0.492 ^*P*=0.006^	-0.537 ^*P*=0.002^
40:5	-0.438 ^*P*=0.001^	-0.489 ^*P* <0.001^	-0.430 ^*P*=0.036^	-0.455 ^*P*=0.019^	-0.464 ^*P*=0.010^	-0.510 ^*P*=0.003^
40:6	-0.420 ^*P* =0.002^	-0.394 ^*P*=0.002^	-0.421 ^*P*=0.040^	-0.302	-0.440 ^*P*=0.015^	-0.379 ^*P*=0.035^
Total PI	-0.478 ^*P* <0.001^	-0.551 ^*P* <0.001^	-0.348	-0.554 ^*P*=0.003^	-0.553 ^*P*=0.002^	-0.556 ^*P*=0.001^

CRP (*p* = 0.747) and fecal calprotectin (*p* = 0.279) did not differ between sexes (Table 1).

**Fig. 2:**
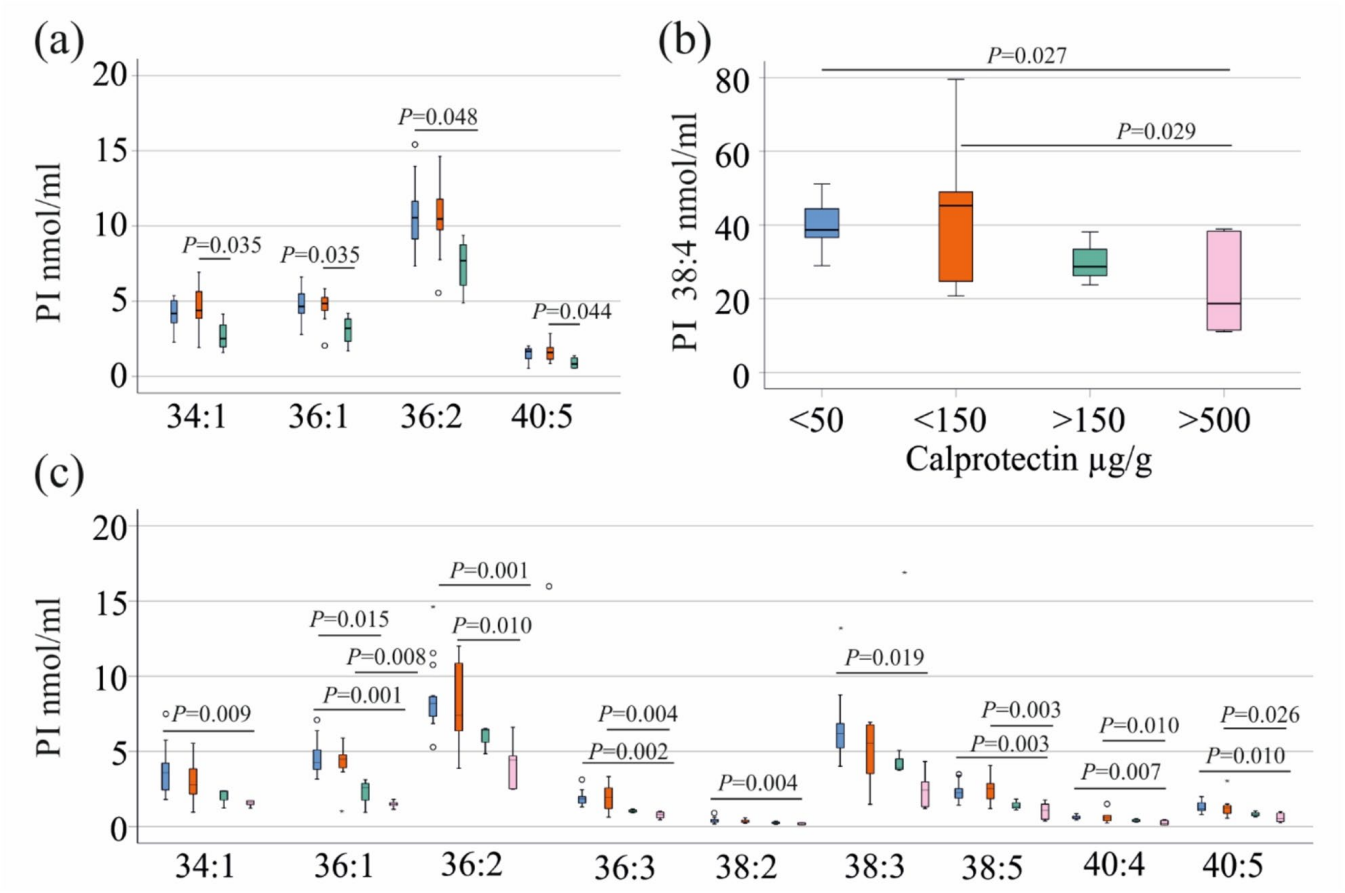
Association of phosphatidylinositol (PI) species with fecal calprotectin levels. **a** PI species showing a decline with increasing fecal calprotectin levels in female patients. Groups: <50 µg/g (blue; *n* = 12), 50–150 µg/g (orange; *n* = 9), 150–500 µg/g (green; *n* = 4); one patient with fecal calprotectin > 500 µg/g is not shown. Statistical test: one-way ANOVA with post-hoc Dunnett. **b** PI 38:4 levels in male patients stratified by fecal calprotectin levels. Statistical test: one-way ANOVA with post-hoc Dunnett. **c** PI species showing a decline with increasing fecal calprotectin levels in male patients. Groups: <50 µg/g (blue; *n* = 15), 50–150 µg/g (orange; *n* = 6), 150–500 µg/g (green; *n* = 4), > 500 µg/g (pink; *n* = 6). Statistical test: one-way ANOVA with post hoc Dunnett’s test, Kruskal-Wallis test with post hoc Bonferroni correction for PI 36:1

In female patients, PI 34:1, 36:1, 36:2, and 40:5 declined with higher fecal calprotectin levels (Fig. 2a). The four patients with fecal calprotectin levels above 150 µg/g had lower levels of PI 34:1, 36:1, and 40:5 compared to the the 9 females with calprotectin levels between 50 and 150 µg/g (Fig. 2a). PI 36:2 levels of 12 females with low (fecal calprotectin < 50 µg/g), and females with fecal calprotectin levels above 150 µg/g were also different. Only one female patient had calprotectin levels above 500 µg/g, and data were not included in the analysis.

In line with the negative correlation between most PI species and fecal calprotectin levels in males (Table 3), total PI levels of the six male patients with high fecal calprotectin (> 500 µg/g) were lower than those of the 15 males with fecal calprotectin levels below 50 µg/g (*P* = 0.003). The PI species which differed were PI:34:1, 36:1, 36:2, 36:3, 38:2, 38:3, 38:4, 38:5, 40:4 and 40:5 (Fig. 2b, c). PI 36:1 in male patients with calprotectin levels between 150 and 500 µg/g was reduced compared to patients with low calprotectin levels (Fig. 2c). The differences in PI 36:1, 36:2, 36:3, 38:5, 40:4, and 40:5 levels between patients with calprotectin between 50 and 150 µg/g and those with levels > 500 µg/g were also significant (Fig. 2c).

Multiple linear regression showed that fecal calprotectin (*P* < 0.001) but not sex (*P* = 0.113) is a significant predictor of total PI levels in serum. Therefore, associations of fecal calprotectin levels with PIs were also analysed in the entire cohort, and all PI species as well as the total PI level of the patients with calprotectin levels below 50 µg/g (27 patients) and of patients with calprotectin between 50 and 150 µg/g (15 patients) were lower in comparison to patients with fecal calprotectin > 500 µg/g (7 patients). PI 36:1 and 40:6 also differed between patients with calprotectin levels < 50 µg/g and those with levels between 150 and 500 µg/g, and between patients with fecal calprotectin of 50–150 µg/g and those with levels of 150–500 µg/g (Additional File 2).

Total PI levels of patients with high fecal calprotectin (> 500 µg/g) were reduced in comparison to healthy controls, patients with levels < 50 µg/g and those with calprotectin levels between 50 and 150 µg/g (Additional File 3). Such differences also existed for phosphatidylcholine, a highly abundant phospholipid class in human serum (Additional File 3). Phosphatidylethanolamine of patients with high fecal calprotectin were reduced in comparison to patients with levels < 50 µg/g and those with calprotectin levels between 50 and 150 µg/g, but did not significantly differ between controls and patients with calprotectin levels > 500 µg/g (Additional File 3).

The Hedges’ g effect sizes were analysed between all patients with calprotectin levels < 150 µg/g and all patients with levels > 150 µg/g fecal calprotectin. Effect sizes were low and only PI 36:1 had a value of 0.5, regarded as a moderate effect size (Table 4). The areas under the ROC curve were accordingly below 0.5, corresponding to a random change [39] (Table 4).

**Table 4:** The hedges’ g effect sizes and areas under the receiver operating curves (ROC) of patients with calprotectin levels below 150 µg/g and those with higher levels

PI Species	Hedges’ g effect size	Area under the ROC curve
	Entire Cohort (*N* = 57)	
34:1	0.1469	0.117
34:2	0.1560	0.190
36:1	0.4999	0.061
36:2	0.1594	0.100
36:3	0.1585	0.094
36:4	0.1175	0.158
38:2	0.1251	0.124
38:3	0.2433	0.133
38:4	0.1111	0.224
38:5	0.1348	0.143
38:6	0.1204	0.150
40:4	0.2673	0.168
40:5	0.1136	0.111
40:6	0.1051	0.172
Total PI	0.1497	0.126

### Correlation of PI species with serum cholesterol

Total serum cholesterol levels in both male and female participants were similar (*P* = 0.345). Notably, the decline of serum cholesterol with higher calprotectin levels was significant in male cases (*P* = 0.004) but not in female patients (*P* = 0.143) [28].

In the entire cohort and in male cases, all PI species and total PI levels showed a strong correlation with cholesterol, which was negatively associated with CRP and calprotectin levels (Table 5). Correlations of total PI level and PI species with CRP or calprotectin (Table 3) were not significant when adjusted for cholesterol serum levels (*P* > 0.05 for all).

In female cases, eight PI species were positively correlated with cholesterol, which showed a negative correlation with fecal calprotectin (Table 5).

**Table 5:** Correlation coefficients for the association between PI species and cholesterol in the total cohort and stratified by sex. Statistical test: pearson’s correlation was used for all but PI 36:1 and 40:6, for which spearman’s correlation was analyzed. The *P*-values in the table were not corrected for multiple comparisons

PI	Cholesterol		
	Entire Cohort (*N* = 57)	Females (*N* = 26)	Males (*N* = 31)
34:1	0.500 ^*P* <0.001^	0.267	0.644 ^*P* <0.001^
34:2	0.470 ^*P* <0.001^	0.347	0.547 ^*P* =0.001^
36:1	0.613 ^*P* <0.001^	0.437 ^*P* =0.026^	0.765 ^*P* <0.001^
36:2	0.582 ^*P* <0.001^	0.501 ^*P* =0.009^	0.643 ^*P* <0.001^
36:3	0.498 ^*P* <0.001^	0.271	0.653 ^*P* <0.001^
36:4	0.570 ^*P* <0.001^	0.428 ^*P* =0.029^	0.678 ^*P* <0.001^
38:2	0.531 ^*P* <0.001^	0.300	0.755 ^*P* <0.001^
38:3	0.636 ^*P* <0.001^	0.480 ^*P* =0.013^	0.736 ^*P* <0.001^
38:4	0.707 ^*P* <0.001^	0.695 ^*P* <0.001^	0.704 ^*P* <0.001^
38:5	0.504 ^*P* <0.001^	0.437 ^*P* =0.025^	0.552 ^*P* =0.001^
38:6	0.486 ^*P* <0.001^	0.374	0.614 ^*P* <0.001^
40:4	0.680 ^*P* <0.001^	0.627 ^*P* =0.001^	0.705 ^*P* <0.001^
40:5	0.598 ^*P* <0.001^	0.509 ^*P* =0.008^	0.651 ^*P* <0.001^
40:6	0.649 ^*P* <0.001^	0.379	0.698 ^*P* <0.001^
Total PI	0.724 ^*P* <0.001^	0.650 ^*P* <0.001^	0.778 ^*P* <0.001^
CRP	-0.395 ^*P =0.003*^	-0.180	-0.504 ^*P =0.005*^
Calprotectin	-0.444 ^*P =0.001*^	-0.425 ^*P <0.031*^	-0.498 ^*P =0.004*^

After adjusting for cholesterol levels, the following associations between fecal calprotectin and PIs were observed: PI 34:1 (*r* = -0.511, *P* = 0.013), PI 34:2 (*r* = -0.551, *P* = 0.006), PI 36:1 (*r* = -0.572, *P* = 0.004), PI 36:2 (*r* = -0.595, *P* = 0.003), PI 36:3 (*r* = -0.489, *P* = 0.018), and total PI levels (*r* = -0.491, *P* = 0.017).

Additionally, PI 36:3 (*r* = -0.433, *P* = 0.039), PI 38:6 (*r* = -0.433, *P* = 0.039), and PI 40:5 (*r* = -0.493, *P* = 0.017) were correlated with CRP after adjusting for cholesterol.

### Association of phosphatidylinositol species with liver enzymes

In female IBD patients, all PI species except PI 38:2 and PI 40:4 showed positive correlations with aspartate aminotransferase (AST) levels. In males, PI 36:1, 38:2, 38:3, 38:4, and 40:4 were positively associated with AST. PI 38:6 correlated with gamma-glutamyl transferase (GGT) in females, while PI 36:4, 38:2, 38:3, and 40:4 correlated with GGT in males. No significant correlations were observed with alanine aminotransferase, alkaline phosphatase or bilirubin (Table 6).

**Table 6:** Correlation of PI species and total PI levels with laboratory parameters of liver function in IBD patients. Statistical test: pearson’s correlation was used for all but PI 36:1 and 40:6, for which spearman’s correlation was analyzed. The *P*-values in the table were not corrected for multiple comparisons

PI Species	AST	ALT	GGT	AP	Bilirubin	AST	ALT	GGT	AP	Bilirubin
	Females (*N* = 26)					Males (*N* = 31)				
34:1	0.529 ^*P* =0.024^	0.235	0.028	0.112	0.055	0.303	-0.006	0.365	-0.030	0.241
34:2	0.521 ^*P* =0.027^	0.112	-0.105	0.207	0.197	0.376	0.176	0.288	0.067	0.226
36:1	0.600 ^*P*=0.009^	-0.040	-0.160	-0.128	0.197	0.318 ^*P* =0.045^	0.058	0.052	-0.011	0.269
36:2	0.654 ^*P* =0.003^	0.049	-0.278	0.045	0.282	0.420	0.172	0.347	0.103	0.262
36:3	0.657 ^*P* =0.003^	0.084	-0.218	0.177	0.269	0.370	0.197	0.367	0.021	0.222
36:4	0.504 ^*P* =0.033^	0.014	-0.062	0.319	0.191	0.371	0.201	0.407 ^*P* =0.032^	0.080	0.196
38:2	0.159	-0.114	-0.185	-0.044	0.090	0.458 ^*P* =0.032^	0.190	0.412 ^*P* =0.030^	-0.023	0.190
38:3	0.542 ^*P* =0.020^	0.070	-0.133	0.310	0.098	0.520 ^*P* =0.013^	0.318	0.464 ^*P* =0.013^	0.006	0.169
38:4	0.592 ^*P* =0.010^	-0.022	-0.269	0.189	0.020	0.474 ^*P* =0.026^	0.228	0.354	0.141	0.209
38:5	0.629 ^*P* =0.005^	-0.045	-0.237	0.222	0.180	0.228	-0.021	0.235	0.065	0.211
38:6	0.623 ^*P* =0.006^	-0.280	-0.451 ^*P* =0.027^	-0.268	0.106	0.236	0.025	0.097	0.071	-0.041
40:4	0.449	0.107	-0.032	0.309	-0.101	0.451 ^*P* =0.035^	0.158	0.431 ^*P* =0.022^	0.046	0.293
40:5	0.728 ^*P* =0.001^	0.009	-0.340	0.050	0.069	0.375	0.093	0.336	0.049	0.376
40:6	0.414	-0.189	-0.496 ^*P* =0.014^	-0.221	0.210	0.284	-0.014	-0.256	-0.067	0.204
Total PI	0.664 ^*P* =0.003^	0.007	-0.258	0.178	0.095	0.484 ^*P* =0.022^	0.213	0.413 ^*P* =0.029^	0.097	0.239

Alanine aminotransferase (*ALT*), alkaline phosphatase (*AP*), aspartate aminotransferase (*AST*), gamma-glutamyl transferase (*GGT*)

### Comparison of PI species of patients with CD and UC

**Fig. 3:**
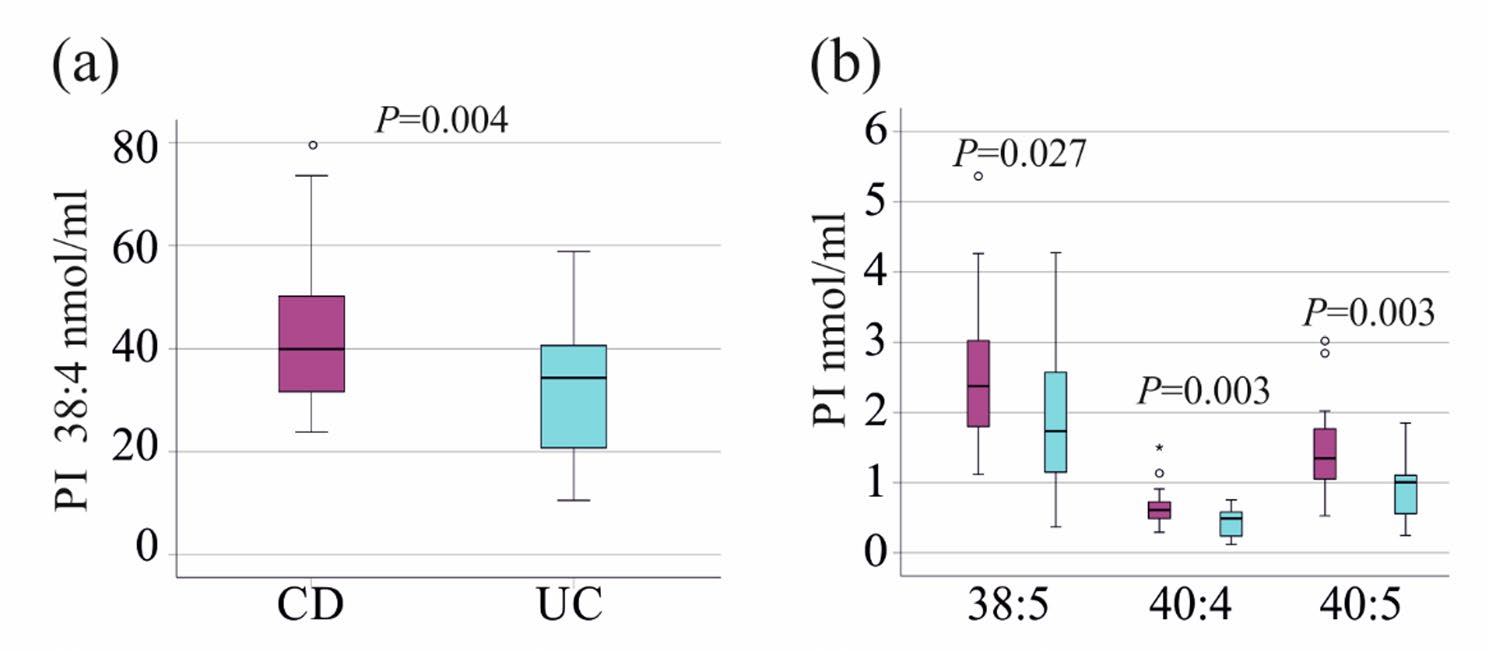
PI species, which differ between patients with Crohn´s disease (CD) and ulcerative colitis (UC). **a** PI 38:4 in the serum of the 40 patients with CD and the 17 patients with UC. **b** PI 38:5, 40:4 and 40:5 in the serum of the 40 patients with CD and the 17 patients with UC. Statistical test: One-way ANOVA. The *P*-values in the figures were not corrected for multiple comparisons

PI 38:4, 38:5, 40:4 and 40:5 differed between patients with CD and UC, and were reduced in UC (Fig. 3a, b). These patients had similar age, BMI, calprotectin and CRP levels. Total serum cholesterol was also comparable (*P* > 0.05 for all). There were 21 females in the CD cohort (40 patients) and 5 females in the UC cohort (17 patients) with a similar distribution of sexes.

Receiver operating characteristic curve showed an area under the curve (AUROC) of 0.721 ± 0.076 for PI 38:4 (*p* = 0.009), 0.678 ± 0.084 for PI 38:5 (*P* = 0.035), 0.726 ± 0.075 for PI 40:4 (*P* = 0.007), and 0.754 ± 0.071 for PI 40:5 (*P* = 0.003) to discriminate CD from UC.

### Disease localisation and association with serum PI species

In patients with CD, isolated ileocecal involvement was observed in eight patients (seven females, one male), while 29 patients showed ileocecal involvement combined with additional gastrointestinal regions (11 females, 18 males). Three female patients had no ileocecal involvement. Disease localisation differed significantly between sexes (*P* = 0.011). However, serum phosphatidylinositol (PI) species levels did not differ significantly across these subgroups in the total cohort (*P* > 0.05 for all comparisons). Due to limited subgroup sizes, sex-stratified analysis was not performed.

**Fig. 4:**
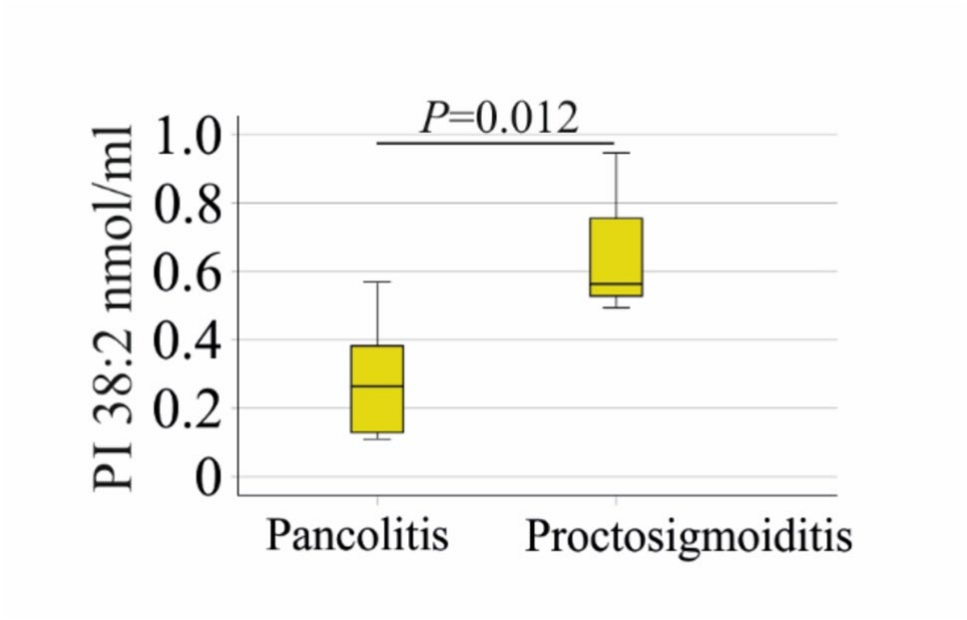
PI 38:2 in relation to disease localization in patients with ulcerative colitis. PI 38:2 in the 10 patients with pancolitis compared to the 3 patients with proctosigmoiditis. Statistical test: One-way ANOVA. The *P*-value in the figure was not corrected for multiple comparisons

Among patients with ulcerative colitis (UC), one male presented with isolated proctitis, while one female and two males had proctosigmoiditis. Left-sided colitis was observed in two males, and pancolitis in eight males and two females. In one patient, disease localisation was undocumented. Disease distribution did not differ between female and male UC patients (*P* = 0.874). Patients with proctosigmoiditis showed higher serum PI 38:2 levels (0.56 (0.49–0.95) nmol/ml) compared to those with pancolitis (0.26 (0.11–0.57) nmol/ml; *P* = 0.012; Fig. 4).

Age (*P* = 1.000), serum cholesterol (*P* = 0.176), CRP (*P* = 0.610) and fecal calprotectin (*P* = 0.128) of patients with pancolitis and proctosigmoiditis were similar.

## Discussion

This study demonstrates that the majority of the 14 analysed PI species were inversely associated with C-reactive protein and fecal calprotectin levels in male patients with IBD, while correlations in females were primarily observed with fecal calprotectin. Given the known anti-inflammatory properties of PI species [29], reduced PI levels in IBD may contribute to ongoing intestinal inflammation and disease activity. Serum PI species levels in patients with IBD were comparable to those in healthy controls, suggesting a very limited diagnostic value for distinguishing IBD from controls. Although reductions in PI species were observed in patients with active inflammation, the magnitude of change appears insufficient for reliable monitoring of disease activity.

To our knowledge, PI levels in the serum of patients with IBD have not been studied in detail, and only a few studies have analysed this lipid class in mice. Fecal phospholipids are mostly derived from bile, shed epithelial cells, and intestinal bacteria. Mice that were given azoxymethane and dextran sodium sulfate to induce colitis-associated tumors had lower levels of PIs in their stool than control animals [40]. This suggests that a decline in serum PI levels during active colitis may result in lower PI levels in the colon and ultimately in feces. Experimental studies have shown that intraperitoneal administration of PI in 2,4,6-trinitrobenzene sulfonic acid-induced colitis reduces T cell-derived inflammatory cytokine release and improves histological inflammation scores [29]. These findings are consistent with the inverse correlations between serum PI species and fecal calprotectin observed in our study, suggesting a potential anti-inflammatory role of circulating PI in intestinal inflammation.

Interestingly, most of the PI species were found to be negatively correlated with CRP and fecal calprotectin in male patients: In females most of the PI species were negatively correlated with fecal calprotectin, with only three PI species associated with CRP. This suggests that serum levels of PIs in females are more closely related to mucosal than to systemic inflammation.

Serum cholesterol levels were inversely correlated with CRP and fecal calprotectin in male patients, while in females, correlations were restricted to fecal calprotectin. A significant reduction in cholesterol was observed in males with active IBD [28]. In males, all associations between PI species and fecal calprotectin disappeared after adjustment for cholesterol, likely reflecting shared links between lipid metabolism and inflammation. In contrast, after adjusting for cholesterol, negative associations between several PI species (PI 34:1, 34:2, 36:1, 36:2, 36:3 with fecal calprotectin; PI 36:3, 38:6, 40:5 with CRP) remained significant in females. These findings highlight that PI species, while being constituents of lipoproteins [41], reflect inflammatory activity also independently of cholesterol, particularly in female patients. However, the decline of total PI, along with lower levels of cholesterol and additional phospholipid classes such as phosphatidylcholines and phosphatidylethanolamine in patients with active IBD, suggests a close association between inflammation and hypolipidemia [18].

PI has been shown to promote cellular cholesterol efflux to HDL, thereby increasing HDL cholesterol levels in humans [10–12]. Reduced serum PI levels in patients with active IBD may therefore contribute to hypocholesterolaemia observed in inflammation. However, the temporal relationship between declining PI and cholesterol levels in the context of intestinal inflammation remains to be elucidated.

Impaired transfer of cellular cholesterol to lipoproteins can lead to the accumulation of cholesterol in macrophages, which can become foam cells. This may contribute to an increased risk of atherosclerosis in patients with IBD [42]. HDL also has anti-inflammatory properties, and lower levels may contribute to cardiovascular disease [43].

Serum PI levels were increased in female compared to male patients with IBD, and nine PI species were significantly higher in females. There was no difference in serum cholesterol levels between the sexes, excluding variations in this lipid class as the underlying reason. Among the control group, two of these PI species were higher in females than in males, and the small size of this cohort may have prevented the identification of further differences. Although our study indicates a sex-specific difference of PI levels in serum, in a group of ten healthy females and ten healthy males aged 25, serum PI species levels did not differ between the sexes [44]. Further studies are needed to clarify the association between PI species and sex.

Twelve PI species in female patients´serum, and five in male patients´serum were positively correlated with aspartate aminotransferase levels, which were mostly within the normal range. The median levels of other laboratory tests for liver disease were also normal, suggesting that most of our patients had healthy livers. Previous studies have reported that PI levels in the blood of patients with MASLD can be normal or elevated. However, it remains unclear whether specific PI species are correlated with the severity of liver disease [45, 46, 47]. Up to 50% of patients with IBD have elevated liver enzyme levels, and the common causes are underlying metabolic dysfunction-associated steatotic liver disease, drug toxicity, and primary sclerosing cholangitis [48]. Patients with primary sclerosing cholangitis were excluded from the current study. The positive correlation between PI species and aspartate aminotransferase, but not additional liver disease measures such as bilirubin, suggests that PI levels are not associated with liver function in patients with IBD. It has been shown that dietary PI protects against liver injury by modulating the function of immune cells and inflammation [49, 50]. Based on this finding, it would be expected to find a negative correlation between serum PI levels and measures of liver disease.

In our cohort, disease localization differed by sex in patients with CD but not in patients with UC. A recent analysis found that men were less likely to have proctitis and more likely to have extensive colitis [51]. Furthermore, it was demonstrated that, compared to females, the upper gastrointestinal tract and the ileal region are most often affected in male patients with CD [52]. The small cohort size limits conclusions regarding the lower frequency of isolated ileocecal Crohn’s disease in males. Serum PI species levels did not differ by disease localisation in CD. Due to the limited sample size, sex-stratified analysis was not feasible.

Proctosigmoiditis affects the rectum and the sigmoid colon. Pancolitis is a severe form of UC affecting the entire colon and rectum [53]. The three patients with proctosigmoiditis had approximately twice the level of PI 38:2 compared to all other patients with UC. Markers of inflammation and cholesterol levels in patients with UC and different disease localisations were similar, so these can be excluded as confounding factors. However, the number of patients with proctosigmoiditis was small, so this result must be confirmed in much larger cohorts.

This exploratory study further provides preliminary evidence that PI 38:4, 38:5, 40:4, and 40:5 levels differ between patients with CD and UC. Again, due to limited cohort size, sex-stratified analysis could not be performed. As it can still be difficult to distinguish between CD and UC in some patients [54], additional diagnostic biomarkers may be helpful.

Egg yolk, a rich source of PI, alleviated intestinal inflammation in murine colitis models [55]. While soybeans exhibit anti-inflammatory effects in IBD, the contribution of their high PI content remains unclear [56]. Further studies are warranted to clarify whether dietary PI supplementation can modulate intestinal inflammation in IBD.

This study has limitations. The laboratory values and body mass index of the control group were not recorded. The small size of the study cohorts also made subgroup analysis difficult. The findings of this exploratory study must be confirmed by future research.

In conclusion, this exploratory study is the first to demonstrate that PI species decline in active IBD, and further dedicated studies are required to validate these results.

## Supplementary Information

Supplementary Material 1.

Supplementary Material 2.

Supplementary Material 3.

## Data Availability

Original research data can be obtained upon request.

## Abbreviations

ALT: Alanine aminotransferase
AP: Alkaline phosphatase
AST: Aspartate aminotransferase
CD: Crohn´s Disease
GGT: Gamma-glutamyl transferase
HDL: High-density lipoprotein
IBD: Inflammatory Bowel Disease
LDL: Low-density lipoprotein
PI: Phosphatidylinositol
UC: Ulcerative Colitis
