# Supplementary material for: Serum phosphatidylinositol depletion associates with fecal calprotectin and disease severity in female and male IBD patients
Source: Lipids Health Dis. 2026 Feb 4;25:67. doi: 10.1186/s12944-026-02889-3 (PMC12930587; doi:10.1186/s12944-026-02889-3)
Supplement: Supplementary file 1 — Supplementary Material 1. [file 12944_2026_2889_MOESM1_ESM.pdf]

Created by https://lipidomicstandards.org, version v2.5.0

Contents of Report

Direct Infusion Workflow 1
- Overall study design 1
- Lipid extraction 1
- Analytical platform 1
- Quality control 1
- Method qualification and validation 2
- Reporting 2

Sample Descriptions 2
- IBD Serum / Human / Serum 2

Lipid Class Descriptions 2
- 1) PI[M+NH4]+ / Lipid identification 2
- 1) PI[M+NH4]+ / Lipid quantification 2

Direct Infusion Workflow

Overall study design

Title of the study	PI Quantification (FIA-QQQ)		
Document creation date	09/26/2025	Principal investigator	Gerhard Liebisch
Institution	Institute of Clinical Chemistry and Laboratory Medicine, University Hospital Regensburg	Corresponding Email	gerhard.liebisch@ukr.de
Is the workflow targeted or untargeted?	Targeted	Clinical	No

Lipid extraction

Extraction method	2-phase system	pH adjustment	None
2-phase system	Bligh&Dyer	Were internal standards used?	Yes
Deposition method	NA	Internal standards used	yes

Analytical platform

Ionizaton additives	Ammonium acetate	Detector	Mass spectrometer
MS type	QQQ	MS vendor	Waters
Direct type	FIA	MS Level	MS^2
Mass window for precursor ion isolation (in Da total isolation window)	0.8	Mass resolution for detected ion at MS^2	Low resolution
Resolution at MS^2	Unit	Recording mode of raw data at MS^2	Profile mode
Was/Were additional dimension/techniques used	No		

Quality control

Blanks	Yes	Type of Blanks	Solvent blank, Internal standard blank
Quality control	Yes	Type of QC sample	Sample pool

Method qualification and validation

Method validation	Yes	Lipid recovery	Yes
Dynamic quantification range	No	Limit of quantitation (LOQ)/Limit of detection (LOD)	Yes
Precision	Yes	Accuracy	Yes
Guidelines followed	None		

Reporting

Are reported raw data uploaded into repository?	Available on request	Are metadata available?	Available on request
Raw data upload	Available on request		

Sample Descriptions

IBD Serum / Human / Serum

Storage and collection conditions	Available	Temperature handling original sample	Room temperature
Instant sample preparation	No	Storage temperature	-80 °C
Additives	None		

Lipid Class Descriptions

1) PI[M+NH4]+ / Lipid identification

Lipid class	PI	MS Level for identification	MS^2
Identification level	Species level	MS^2 adduct	[M+NH4]+

Fragments for identification

Fragment name
-HG(PI,277)

Isotope correction at MS^2	Type 2	MS^2 verified by standard	Yes
Background check at MS^2	Yes	Did you presume assumptions for identification?	Yes
Which assumptions were presumed?	Presence of acyl-bond for all species	Check on:	Isomeric overlap, Isobaric overlap
Limit of detection	Signal theshold	Lipid Identification Software	Homemade
Data manipulation	Smoothing, Centroiding, Background subtraction	Nomenclature for intact lipid molecule	Yes
Nomenclature for fragment ions	N/A		

1) PI[M+NH4]+ / Lipid quantification

Quantitative	Yes	MS Level for quantification	MS^2

Internal lipid standard(s) MS^2

Internal standard	Fragment(s)	Endogenous subclass
PI 15:0/18:1[D7]	-HG(PI,277)	all PI species

Type of quantification	Internal standard amount	Response correction	No
Type I isotope correction	Yes	Limit of quantification	Signal theshold
Normalization to reference	Yes	Lipid Quantification Software	Homemade
Batch correction	No		
