# Supplementary material for: Serum phosphatidylinositol depletion associates with fecal calprotectin and disease severity in female and male IBD patients
Source: Lipids Health Dis. 2026 Feb 4;25:67. doi: 10.1186/s12944-026-02889-3 (PMC12930587; doi:10.1186/s12944-026-02889-3)
Supplement: Supplementary file 2 — Supplementary Material 2. [file 12944_2026_2889_MOESM2_ESM.pdf]

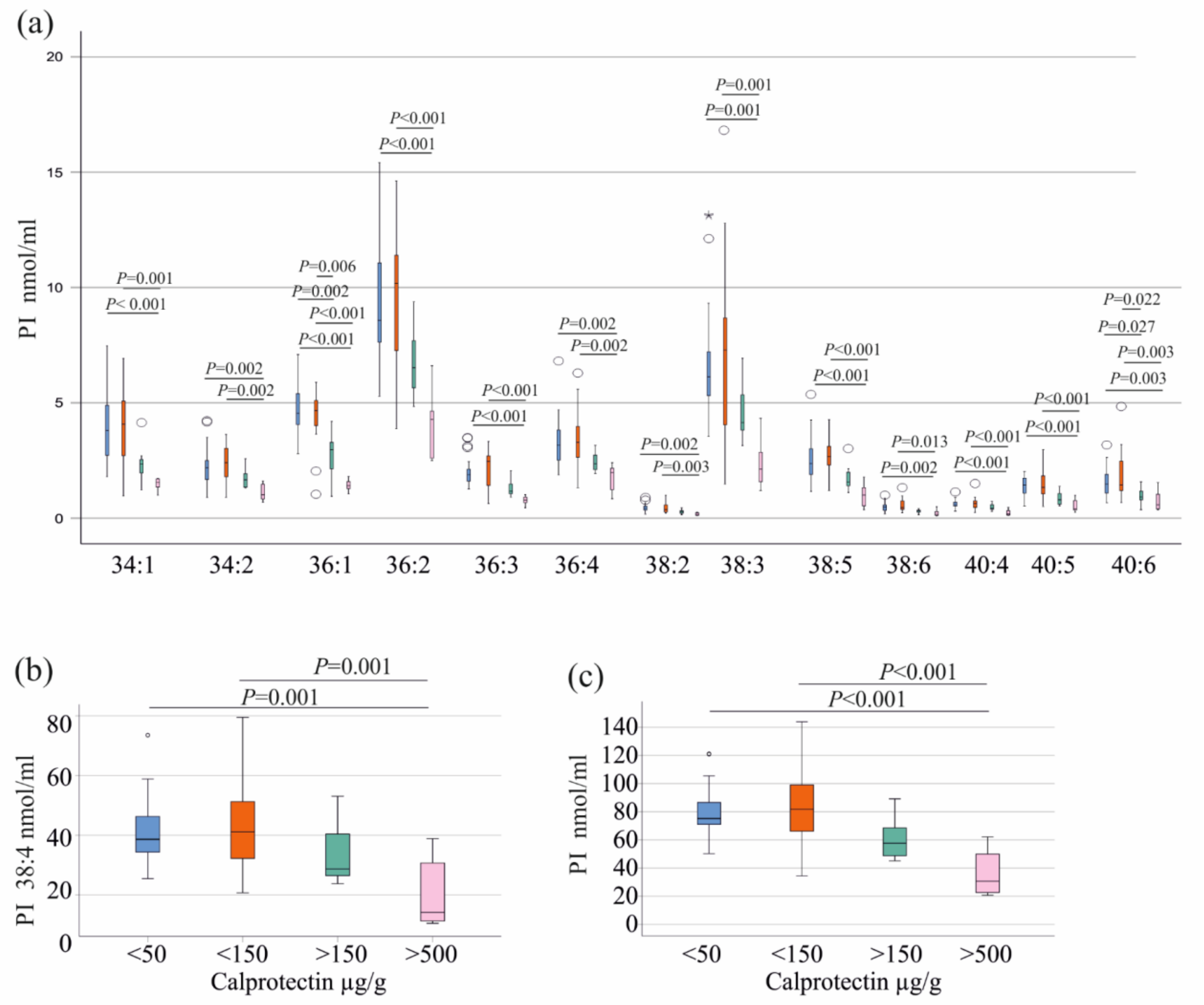

Additional File 2. Association of phosphatidylinositol (PI) species with fecal calprotectin levels in the entire cohort. (a) PI species showing a decline with increasing fecal calprotectin levels in the entire patient cohort. Groups: <50 µg/g (blue; n = 27), 50–150 µg/g (orange; n = 15), 150–500 µg/g (green; n = 8); >500 µg/g (pink; n = 7). (b) PI 38:4 levels in the patients stratified by fecal calprotectin levels. (c) Total PI levels in the entire cohort. Statistical test: one-way ANOVA with post hoc Dunnett's test, Kruskal–Wallis test with post hoc Bonferroni correction for PI 36:1and 40:6.
