# Supplementary material for: Serum phosphatidylinositol depletion associates with fecal calprotectin and disease severity in female and male IBD patients
Source: Lipids Health Dis. 2026 Feb 4;25:67. doi: 10.1186/s12944-026-02889-3 (PMC12930587; doi:10.1186/s12944-026-02889-3)
Supplement: Supplementary file 3 — Supplementary Material 3. [file 12944_2026_2889_MOESM3_ESM.pdf]

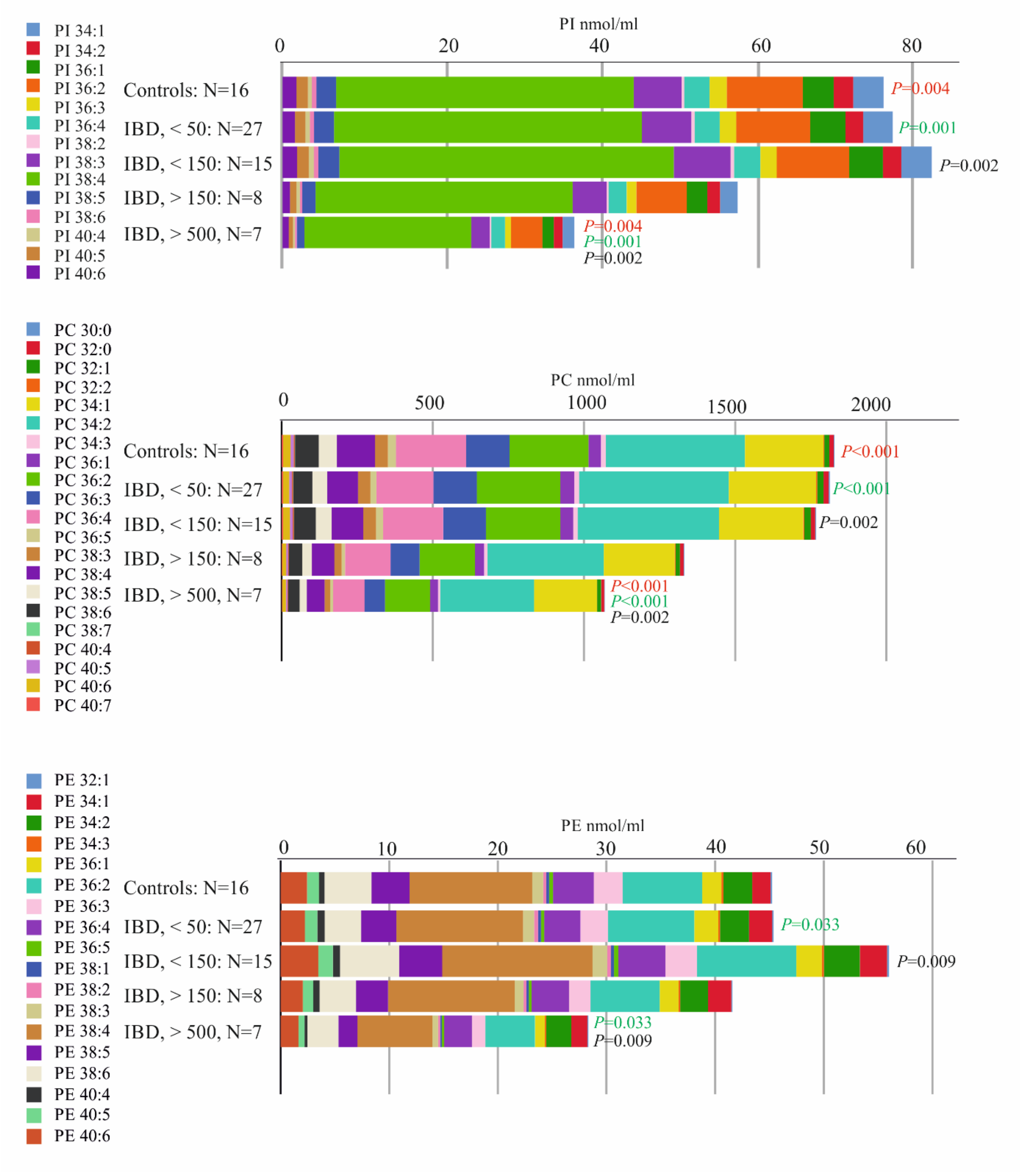

.

Figure S2. Association of phosphatidylinositol (PI), phosphatidylcholine (PC) and phosphatidylethanolamine (PE) levels in serum with fecal calprotectin levels. (a) Total PI levels obtained by summing the measured PI species in the controls (n = 16), patients with IBD and fecal calprotectin <50 µg/g (n = 27), 50–150 µg/g (n = 15), 150–500 µg/g (n = 8) and >500 µg/g (n = 7). (b) Total PC and (c) Total PE levels in these cohorts. Statistical test: one-way ANOVA with post hoc Dunnett's test. The p-values for comparisons between two groups are shown in the same colour. Statistical test: one-way ANOVA with post hoc Dunnett's test.
